# Hypnotic drug risks of mortality, infection, depression, and cancer: but lack of benefit

**DOI:** 10.12688/f1000research.8729.3

**Published:** 2018-11-12

**Authors:** Daniel F. Kripke

**Affiliations:** 1 University of California, San Diego, La Jolla, CA, 92037-2226, USA

**Keywords:** hypnotics and sedatives, mortality, cancer, infection, depression, insomnia, sleep

## Abstract

This is a review of hypnotic drug risks and benefits. Almost every month, new information appears about the risks of hypnotics (sleeping pills). The most important risks of hypnotics include excess mortality (especially overdose deaths, quiet deaths at night, and suicides), infections, cancer, depression, automobile crashes, falls, other accidents, and hypnotic-withdrawal insomnia. Short-term use of one-two prescriptions is associated with even greater risk per dose than long-term use. Hypnotics have usually been prescribed without approved indication, most often with specific contraindications, but even when indicated, there is little or no benefit. The recommended doses objectively increase sleep little if at all, daytime performance is often made worse (not better) and the lack of general health benefits is commonly misrepresented in advertising. Treatments such as the cognitive behavioral treatment of insomnia and bright light treatment of circadian rhythm disorders offer safer and more effective alternative approaches to insomnia.

## Introduction

This is the third update and expansion of scientific review presented October 26, 2015 to the Commissioner of the Food and Drug Administration (United States FDA) as part B of Petition FDA-2015-P-3959. That petition is accessible at http://www.regulations.gov/#!docketDetail;D=FDA-2015-P-3959 along with peer Comments responding to that Petition. Almost every month, new information about the risks of hypnotics (sleeping pills) appears.

## Risks of hypnotic drugs

### Hypnotic drugs increase all-cause mortality

Use of hypnotic drugs is associated prospectively with a greatly increased risk of all-cause mortality. Some of this mortality has been documented as deaths caused by hypnotics by Medical Examiners, attributed to respiratory arrests resulting from “overdose.” However, it is likely that many deaths from respiratory depression occur among patients never examined by coroners, especially when the death is caused by a combination of hypnotics with other contributing factors, so that the lethal hypnotic dosage may by itself have been within currently-customary dosage ranges. In addition to respiratory depression, hypnotics are related to serious illnesses and premature deaths from cancer, serious infections, mood disorders, accidental injuries, suicides and homicides.

### The overdose epidemic

Only a small fraction of U.S. deaths has been medically evaluated by coroners and Medical Examiners. Thus, it is commonly assumed that overdose deaths have been grossly under-reported. Despite such underestimation, U.S. drug and opioid overdose deaths reported in 2014 reached 47,055, a 137% increase since 2000 ^1^, and rapidly increased to 72,000 per year in 2017–2018 ^2^. Self-injury deaths including overdoses that might have been accidental or cause-undetermined are higher ^3^, so total self-injury deaths would be estimated well above 72,000 for 2018. U.S. use of hypnotics dramatically increased over most of the same interval until about 2012 ^4^ but then have subsequently decreased ^5^.

Available death certificate reports seem unclear, but perhaps one third of death certificates listing overdose with an opioid as a cause of death also lists a benzodiazepine, Z hypnotic, or barbiturate as a cause of death (retrieved from CDC Wonder). Such death certificate data are known to underestimate drug involvement, due to insufficient assays done and insensitivity of assays when blood is not frozen soon after death. In about half of overdose reports, the drug causing death has been unspecified ^6^. A very recent study combining Medical Examiner toxicity reports with prescription records suggested that 76% of overdose patients had a benzodiazepine agonist prescription: e.g., one quarter of the patients had a prescription for the hypnotic zolpidem ^7^. Some older studies are consistent with these high estimates of combination overdoses ^8^. An opioid prescription is more likely to lead to overdose when a hypnotic is also prescribed ^9^. Indeed, among women who had received an opioid prescription, the added overdose risk of taking high-dose benzodiazepine or zolpidem was comparable to the risk of high-dose opioid ^10^. It appears that some of the hazard associated with zolpidem was attenuated when confounding depression was included in the statistical adjustment; however, since hypnotics cause depression (see below), hypnotics would seem indirectly causal for much of the depression-related excess hazard ^10, 11^. There have also been several thousand yearly reported overdoses involving a hypnotic in which an opioid was not involved. With both opioid and other-drug poisoning increasing ^12^, so great is the recent increase in overdose and suicide deaths that they have lowered overall life expectancy in much of the U.S. adult population ^13, 14^. Overdoses kill more Americans than automobile accidents or murders. On August 31, 2016, the FDA announced “boxed warnings” about the lethality of combinations of opiates and benzodiazepine agonists including benzodiazepine hypnotics.

Aside from overdose deaths, benzodiazepines were involved in a comparably increasing number of emergency room visits over a similar time interval, and over half of these also involved opioids, alcohol, or all three in combination ^15^. Combined overdoses of opiates and benzodiazepine agonists had more severe outcomes. Suicides from all causes per capita have been increasing, particularly among women, and overdoses from unspecified drugs have been increasing, particularly since 2007, at which time generic zolpidem became available, increasing zolpidem prescribing ^16^. Hypnotics may increase suicide by increasing depression, impairing judgement, and creating behavioral confusion (see below), as well as through pharmacologic overdosage.

There is some evidence that increasing hypnotic use is epidemiologically associated with the social despair and underemployment similar to that associated with the increasing opioid overdose epidemic. This despair is linked to depressed mood, sleep complaints, weakness, and poor motor coordination ^17^.

### Forty-six epidemiologic studies of mortality

Of 46 epidemiologic studies that provided comparable risk ratios for mortality associated with hypnotics, 43 found that hypnotics were associated with excess mortality, as listed in Appendix A. In addition, a 44 ^th^ study of stroke patients showed elevated mortality among those receiving hypnotics and various other psychotropic drugs ^18^. One exception was an early small study by Merlo *et al.* that nevertheless found hypnotics associated with cancer deaths ^19^. Another partial exception was a study from Taiwan that found benzodiazepine hypnotics associated with significant excess mortality, but found zolpidem 10 mg associated with significantly reduced mortality in adjusted models, despite a significantly-increased unadjusted mortality risk for zolpidem and a significantly-increased adjusted risk of cancer mortality for zolpidem ^20^. In a comment to this report appearing with it on the internet, I have questioned the statistical methods of adjusting zolpidem risks ^20^. A large study of benzodiazepine use and mortality was not included because it was not focused on hypnotics, specifically excluded nonbenzodiazepine “Z” drugs such as zolpidem, and failed to compare drug use of cases and controls during follow-ups ^21^.

Only that one of the 46 epidemiologic studies of hypnotic drugs reported any association with improved patient survival, and that one only by relying on questionable statistical adjustments ^20^. None of the other 45 studies found hypnotic drug risk ratios significantly less than 1.0. That is, in 45 studies there was no evidence that hypnotics ever benefit patient survival. To find 44 of 46 studies showing a positive risk ratio was very highly significant, P<0.000001. Also, the evidence of association satisfied all nine Bradford Hill criteria for inferring causality ^22^, though skepticism despite meeting these criteria may be warranted ^23^. There remain questions concerning the magnitude of the causality. The randomized placebo-controlled trials I have suggested would help clarify the magnitude of hypnotic health impairment ^22^.

Of the 46 epidemiologic studies, 35 individual studies reported statistically significant mortality odds ratios, risk ratios, or hazard ratios for hypnotics exceeding 1.0. All 22 studies reporting on samples of >14,000 people found significant mortality risks, but nine of 22 smaller studies found positive trends that were not significant. Most of the non-significant reports were among the earliest 15 published before 2006. Of studies analyzing follow-ups of 8 years or less, 23 of 27 studies reported a significant association, but of studies with longer follow-ups, only 13 of 17 studies observed significant mortality risks. This may suggest that during long prospective follow-ups, many patients initially taking hypnotics will discontinue hypnotic usage, whereas many controls not using hypnotics at prospective baseline may have begun using hypnotics during a long follow-up, so that the longer the follow-up, the more mixing of hypnotic-consuming and control groups becomes likely. Mixing weakens the risk-ratio contrasts observed. In long follow-ups, one is also studying the selected survivors of the more marked short-term risks that have been recently described ^24, 25^.

Most of the 46 studies reported mortality risk ratios of less than 1.5, but some of the highest quality studies reported among the highest risk ratios. Four large studies were particularly persuasive, as presented below.

### The Geisinger Health System study

From electronic records of the Geisinger Health System in Eastern Pennsylvania, a sample of 34,205 patients was drawn with carefully controlled 1:2 matching of hypnotic users with non-user controls for age, gender, smoking, and various comorbidities. Compared to a reference hazard ratio of 1.0 for non-users of hypnotics, the fully-adjusted mortality hazard ratio for use of 0.4–18 hypnotic doses per year was 3.60 (2.92–4.44, 95% CI), for those using 18–132 doses per year, the hazard ratio was 4.43 (3.67–5.36), and for >132 doses per year, the hazard ratio was 5.32 (4.50–6.30) ^26^. Each of these associations was significant with P<0.001. Sensitivity studies showed that little of the hypnotic-associated mortality could be explained by known confounders or use of hypnotics before commencement of the study. In this sample, prescriptions for each of the following drugs were found to significantly predict increased mortality with statistical significance: zolpidem, temazepam, eszopiclone, zaleplon, triazolam, flurazepam or quazepam, and barbiturates prescribed to induce sleep. This review is principally concerned with these popular hypnotics for which drug-specific mortality data are available. Barbiturates prescribed at night for sleep considered as a group had about the same empirical hazard ratios as the benzodiazepines and zolpidem, but the observed hazard ratio for eszopiclone was significantly higher than that of barbiturates, possibly biased by the shorter average follow-up intervals for this more-recently introduced drug ^26^.

### The Weich *et al.* study

In a sample of over 100,000 hypnotic users and matched controls from the representative British General Practice Research Database ^27^, users of 1–30 defined daily doses (DDD) of hypnotics and anxiolytics within a year had fully adjusted dose-responsive mortality hazard ratios of 2.55 (2.42–2.69, 95% CI) for 1–30 DDD (defined daily doses in the first year); 3.78 (3.54–4.04) for 31–60 DDD, 4.19 (3.84–4.58) for DDD 61–90, and 4.51 (4.22–4.82) for DDD >90. Extensive full adjustment for potential confounders resulted in only very small and inconsistent decreases in the estimated hazard ratios, and many methodological details were focused on minimizing possibilities of confounding. Use of benzodiazepine hypnotics alone was associated with higher hazard ratios than use of “Z” hypnotics alone. These hazard ratios were remarkably similar to those from the Geisinger Health System, considering the many differences in drug characteristics, samples, design, confounder controls, and analyses. Note that as in the Geisinger Health System study, much of the mortality was associated with early deaths after limited doses of hypnotics, perhaps as little as one-two prescriptions filled or refilled.

### Norwegian Pharmacy Database

A representative study of the Norwegian Pharmacy Database found that benzodiazepine-receptor-agonist use was associated with a mortality odds ratio of 2.30 (2.20–2.40) ^28^. The authors argued that terminal illness caused an upturn in benzodiazepine-receptor agonist use shortly before death (which might be appropriate for hospice care), and therefore they argued that the increased benzodiazepine-agonist use among those who would die was demonstrated as a confound of terminal illness. To the contrary, their data demonstrated an excess of benzodiazepine use even among those who would not die until 22 months or later, so the benzodiazepine use of this population was elevated before the terminal upturn in hypnotic usage that the authors had demonstrated. Also, the upturn in death-associated hypnotic use 6–10 months before subsequent death might be consistent with a causal lethal hazard resulting from only a few short months' exposures to hypnotics. The Norwegian Pharmacy Database did not enable this study to identify terminal illnesses, to analyze comorbidities or to control for other confounders.

### The Palmaro *et al.* study

In this large study, both French and British case-control samples were drawn from reasonably-representative national samples ^24^. Results had many similarities to those of Weich *et al.* ^27^ despite numerous differences in statistical design. Substantially lower overall hazard ratios were found in the French sample (not all significant after adjustment), perhaps because a large number of occasional users were included. An important finding was the much higher hazard ratios associated with the initial 3–6 months of hypnotics-benzodiazepines use, as high as 11.12 (95% CI, 9.91–12.47) for the 3-month analysis of the British sample. This sharpened the evidence, also noted in the three previous studies discussed, that although dose-response is observed over several years, much of the hypnotic-associated hazard is observed during the early months of usage after as little as one or two prescriptions.

It should be noted that Weich *et al.* ^27^ and Palmaro *et al.* ^24^ found significant hazard ratios associated with diazepam and other benzodiazepines that are not considered hypnotics (though tranquilizer benzodiazepines may often be used for sleep). These more modern data with better drug identification and measurements of prescriptions during follow-up must be considered more reliable, but neither “Valium” nor “Librium” had been associated with excess mortality in the previous large U.S. CPSII study ^29^. One might argue that if diazepam has a different hazard from temazepam, for example, this specificity tends to bolster the evidence for causality with temazepam ^30^. On the other hand, it would not be clear if the specificity is in the drug’s pharmaceutical effects, in its absorption and half-life, in its usual time of administration, in other aspects of frequency and dosage of administration, or in various associated confounders.

These epidemiologic studies had many limitations ^22^. However, the limitations that would tend to bias the results towards underestimating the associations of hypnotics and mortality appeared more influential than those that would bias towards overestimation of the risks. In particular, studies with the most careful efforts to control for confounders found that such control made little difference in the estimated risk ratios, but the hazard ratios in these carefully-controlled studies tended to be higher. The risk ratios derived, like the studies themselves, were extremely heterogeneous, probably due to differences in the size, age, gender, and ethnicity of samples and their health status, the nature of the hypnotics studied, the accuracy with which the drugs examined and their dosages were known, the control variables available, and the duration of follow-up observations to ascertain mortality. Meta-analyses attempting to group 40 such heterogeneous studies would not be clarifying.

### Short-term hypnotic use is unsafe

Data provided by Palmaro *et al.* ^24^ and Chung *et al.* ^25^ expanded the hints in the other large epidemiologic studies ^10, 26– 28^ that short-term hypnotic usage has surprisingly high risks: apparently short-term hypnotic use has higher risks than long-term usage on a per dose or per-unit-time basis. It is logical that for a patient with an “overdose” of common contributory factors such as aging, obesity, sleep apnea, alcohol overuse, and opiate use, even a single hypnotic dose could be lethal on the first night of consumption. Depending on the drug and the patient’s metabolic capacity, the hypnotic drug concentration in blood could increase for the first few consecutive nights of dosage, but eventually, developing tolerance might make each dose less risky among those who had survived the initial doses. There would continue to be deaths at a lower rate after tolerance develops because of hypnotic dose-escalation in response to tolerance, addition of other sedatives or opiates, especially-heavy pre-sleep alcohol consumption, body position, altitude, upper respiratory infections, and other contributing factors that could suddenly produce hypnotic lethality even after several years of steady consumption. In addition, new consumption of non-sedative drugs that impair liver drug metabolism and even foods such as grapefruit can suddenly make a patient more vulnerable to a customary dose. Understanding these considerations, limitation of hypnotic prescribing to a small number of doses or a single prescription cannot be considered safe.

### Are insomnia and depression explanatory confounders?

Several reports carefully examined insomnia and depression as potential confounders of the association of hypnotics with mortality, finding that insomnia and depression could explain little if any of this association ^27, 31– 33^. Note also that the evidence does not permit us to assume that causality between insomnia, depression, and hypnotic usage is a one-way path when contemplating confounder control, as there is reverse causality ^34, 35^.

### Summary of mortality risk epidemiology

Altogether, the epidemiologic literature is conclusive that hypnotic use is associated with excess mortality. The better studies tend to show very high dose-response risk ratios suggesting association with a very large number of deaths. A supplement to the Geisinger Health System data showed that the risk ratios demonstrated lead to estimated U.S. deaths associated with hypnotic usage of the same order of magnitude as those associated with cigarette use, around 300,000–500,000 per year ^26^. Evidence has been presented from several independent studies that most of these deaths cannot be attributed to known forms of confounding, and indeed, adjustment for the major confounders such as smoking and comorbidities produced little change in the estimated associations in most of these studies. Authors acknowledge that their estimates of adjusted association of hypnotics and mortality could be influenced by inadequate ascertainment of confounding factors or lack of control for a very large number of potential confounds with small or rare effects. It is because skeptics may question whether the strong associations of hypnotics with mortality are causal, despite data fulfilling the Bradford Hill criteria for inferring causality ^23^, that large post-marketing controlled trials of vulnerable patients may still be needed ^22^.

### Hypnotic drugs have a long history of delayed recognition of mortality risks

Despite its well-known risks of lethality, pentobarbital was nevertheless for decades a preferred hypnotic routinely prescribed for patients seeking sleep aids. In the U.S., today, the most notable human application of pentobarbital is in implementing the death sentence. Although it has been believed that the more modern benzodiazepine and benzodiazepine-receptor-agonist hypnotics that replaced barbiturates have higher acute margins of safety and therefore lower risks than pentobarbital, death certificate and epidemiologic data do not confirm that the newer drugs are significantly safer than barbiturates in routine use ^22, 36^.

### Hypnotics produce an excess of deaths at night

In the first Cancer Prevention Study, the percentage of deaths at night were found to be increased by 15.6% among those taking hypnotics (P=0.01), presumably due to respiratory suppression ^37^. In that study, the higher percentage of excess deaths at night associated with taking hypnotics accounted for about one third of total excess mortality associated with hypnotics. These nocturnal deaths were attributed to other causes, even though quiet respiratory suppression as a cause would explain the higher percentage of nocturnal deaths observed among those taking hypnotics than among controls.

The mechanisms of dangerous hypnotic respiratory depression are well-understood. The common hypnotics including barbiturates, benzodiazepines, the “Z” drugs and other benzodiazepine-receptor agonists bind to gamma-aminobutyric acid (GABA) receptors. These ligands-agonists alter the configuration of the receptors to allow negative chloride ions to more readily enter the neurons, where the chloride negatively hyperpolarizes the membranes and inhibits the neurons from firing. When they depress neural respiratory center firing, such drugs can acutely suppress respiration and in large enough dosage, or when individuals are particularly sensitive, may effectively arrest respiration, which leads rapidly to cardiac arrest and consequent death ^36, 38, 39^. Respiratory depression is accordingly, and accurately, listed among zolpidem’s warnings and precautions ^40^. The barbiturates and alcohol bind to different locations on GABA receptors, where they exert additive or perhaps synergistic respiratory depression effects which may add to benzodiazepine-agonist effects ^41^. An antihistamine, diphenhydramine, also binds to GABA _A_ receptors, but it does not seem known whether the actions of diphenhydramine on GABA receptors are similar to benzodiazepines. Opiates bind to mu (μ) opioid receptors on respiratory neurons, where they hyperpolarize neural membranes by opening potassium channels ^42^. Thus opiates, benzodiazepine agonists, and alcohol have additive or synergistic effects inhibiting respiratory neurons ^41^. Hypnotics inhibiting respiration would be expected to produce quiet deaths at night.

## Hypnotics can cause serious and potentially lethal infections

A meta-analysis of available placebo-controlled randomized clinical trials showed that hypnotics cause infections (p<0.00001) ^43^. Because these clinical trials randomized hypnotics versus placebos, the 44% higher infection rate among participants who were given hypnotics was proven to be caused by the hypnotics. Moreover, the lead manufacturer of zolpidem has acknowledged that zolpidem induces infections, based on that manufacturer’s own clinical trials data ^44^. The FDA also found dozens of reports of zolpidem-related severe infections among post-marketing reports ^40^.

Extensive epidemiologic data demonstrated that hypnotics are associated with increased pneumonia including fatal pneumonia ^45^. Likewise, triazolam was associated with pneumonia in Japan, perhaps attributable to increased aspiration ^46^. This finding was not confirmed by one Taiwanese study ^47^, but another Taiwanese study focusing on patients with sleep disturbances found that use of zolpidem was associated with 62%–91% increased hospitalizations for serious infections ^48^. A Taiwan study of patients with chronic obstructive pulmonary disease found highly significant odds ratios associated with benzodiazepine use of 9.3 for pneumonia, 10.4 for acute chronic obstructive pulmonary disease (COPD) exacerbation, 45.0 for acute respiratory failure, and 18.6 for cardiopulmonary arrest; whereas the odds ratios for "Z" drugs such as zolpidem were of almost similar magnitude 3 ^25^. In confirmation, note in the Geisinger Health Study supplement, Table 7 ^26^, mortality hazard ratios were likewise specifically elevated among hypnotics users with COPD. Other Taiwanese studies observed that use of zolpidem was associated with increased risk of pyogenic liver abscess ^49^, pancreatitis ^50^, and pyelonephritis ^51^, and zopiclone with pancreatitis and other conditions ^52^. British data showed that use of benzodiazepines and use of the hypnotic zopiclone (containing 50% eszopiclone as the active ingredient) were significantly related to asthma exacerbation and to all-cause mortality following exacerbation ^53^. This asthma study described some of the benzodiazepine-agonist-mediated impairments of immune surveillance ^53^. Perhaps as a consequence of post-hospital continuation of benzodiazepines and resultant infection, use of benzodiazepines was associated with 23% increased hospital readmission in North Carolina ^54^. In summary, epidemiologic evidence indicates that hypnotics not only cause the mild upper-respiratory infections most commonly reported in available controlled clinical trials ^43^, but also more severe and life-threatening infections. Since such infections demonstrably impair survival, infection is shown to be an additional mechanism by which hypnotics covertly increase mortality. The death certificate would be likely to list the infection as a cause of death but not the hypnotic which may have caused that infection.

Animal studies confirm that hypnotics can cause infections. A controlled trial demonstrated in mice that diazepam exacerbated *Streptococcus pneumoniae* infection through GABA _A_ receptors, partly explaining the underlying immune mechanisms ^55^. In mice, diazepam also exacerbated cowpox, a viral infection ^56^. Midazolam impaired equine immune responses, attributable to effects on macrophage peripheral benzodiazepine receptors (now called TSPO) ^57^. Evidence for involvement of the peripheral benzodiazepine receptor TSPO in immune impairment also came from specific test compounds in mice ^58^. Thus, hypnotic drugs cause increased risk of potentially lethal infections in controlled laboratory experiments.

## Hypnotics are associated with increased cancer

### Human clinical trials strongly suggested that hypnotics cause cancer

A compilation of randomized controlled trials of hypnotics showed 12 cancers or tumors of uncertain malignancy reported among participants randomized to a hypnotic, but none (zero) among those randomized to placebo (P=0.032, two-tailed Fisher Exact Test) ^59^. When the FDA repeated this audit of their controlled trials data, they counted 13 cancers among those randomized to hypnotics versus none (zero) from placebo ^59^.

The controlled-trials compilations described above did not include indiplon, an unlicensed zaleplon-like benzodiazepine agonist and hypnotic, for which studies published subsequently indicated three incident cancers in the indiplon groups and none in the randomized control groups ^60, 61^. The compilations did include cancers associated with the marketed hypnotic ramelteon that admittedly has a very different molecular mode of action from the benzodiazepine agonists.

The FDA was not persuaded that these human controlled-trials data required regulatory action, because most of the definite cancers were only minor skin cancers, because of heterogeneities in the data, and because the cancers were recognized after such short randomization periods. Nevertheless, the controlled trials data suggested more than skin cancer. There were cancers of organs apart from skin noted among those treated with hypnotics but none among those randomized to placebo. Reconsideration of FDA's deferral of action is now encouraged by new animal testing and new epidemiologic findings: over half of the research referenced in this manuscript appeared after that FDA deferral of action.

Because hypnotics seem to cause cancers to be suddenly recognized during short clinical trials, e.g., from one month to one year, the short-term effects are likely to arise more from hypnotics promoting progression of tiny pre-existing cancers rather than from effects upon microscopic cancer initiation. Such progression may cause a cancer death, whether or not the hypnotics initiated the cancer.

### Animal studies proved that hypnotics cause cancer

The animal data in the FDA files for zolpidem indicated that increasing doses of zolpidem fed to rats resulted in increasing numbers of renal liposarcomas and lipomas combined (statistically significant). These data also showed increased thyroid follicular adenomas and carcinomas combined, and increased testicular interstitial cell adenomas, but the latter findings did not reach statistical significance ^62^. There were no such tumors – that is, zero tumors – in the placebo groups. These studies were too small, however, to have substantial power for these neoplasms. Expert FDA pharmacy examiners interpreted the data as suggesting an unknown degree of cancer risk for humans.

These experiments, which showed tumors resulting from feeding zolpidem to rats and suggested a dose-dependent relationship, apparently were never extended, clarified, published, or otherwise followed up.

Similarly, the animal data used for eszopiclone evaluation relied largely on old zopiclone data, since eszopiclone is roughly 50% of zopiclone, and eszopiclone is thought to be the active isomer. Along with other issues, the animal evidence that zopiclone caused animal cancers was of great enough concern to FDA’s scientists, that at least five FDA scientists and medical officers recommended against approval of eszopiclone ^63^. Tumors of the lung in rodents were of special concern; these findings also anticipated the human-specific association of hypnotics with lung and esophageal cancers, as will be described below. Despite the cancer evidence and the recommendations of its own experts, the FDA nevertheless approved eszopiclone as a hypnotic.

Since zolpidem and eszopiclone were evaluated, much additional evidence has appeared relating hypnotics to cancer. Amerio *et al.* systematically surveyed FDA records including much animal data not included in the earlier compilation of hypnotics trials and concluded that hypnotics and sedatives had among the most elevated cancer hazards among psychotropic drugs ^64^.

### *In vitro* studies strongly suggest that hypnotics cause cancer

Hypnotics can damage chromosomes. Zopiclone, zaleplon, and ramelteon are clastogenic ^63, 65, 66^. Clastogens are potentially mutagenic agents that induce disruption or breakages of chromosomes. This process can lead to carcinogenesis. Cells that are not killed by the clastogenic effect may become transformed to cancer ^67^. One of the several formulations of zolpidem was said from *in vitro* studies not to be clastogenic ^68^. Other than the four drugs mentioned, no information has been located that other hypnotic drugs found to be associated with cancer have ever been adequately tested for clastogenicity. Clastogenicity is one mechanism by which hypnotics are likely to be carcinogenic, through either initiating cancers or promoting progression through additional mutations of cancer cells, or both.

The alterations of immune surveillance produced by benzodiazepine agonists, discussed in relation to infection above, suggest additional mechanisms by which cancer initiation and progression might be facilitated or disinhibited ^69^. Hypnotic-initiated increases in infections and consequent inflammation is another potential carcinogenic mechanism. These animal-demonstrated and *in-vitro* mechanisms for carcinogenicity of hypnotics, that have been widely ignored, support evidence that hypnotics cause human cancer.

### Human epidemiology studies demonstrate elevated cancer incidence associated with hypnotics

A 2008 paper ^59^ listed three prior epidemiologic studies reporting associations of hypnotics with cancer deaths ^19, 70, 71^. Analysis of CPSII data found that the elevation in deaths associated with hypnotics was comparable to that associated with cigarettes ^71^, though not entirely due to cancer. Mallon, Broman, and Hetta found a much higher cancer adjusted hazard ratio for habitual sleeping pill use of 5.3 (95% C.I. 1.8–15.4) than for smoking among males; none of the specific causes of death were individually significant among females ^70^. A similar result was shown in a later paper for males, but the simple significant mortality elevation of regular hypnotic use among females was lost after multivariate adjustment in the second study ^32^. More recently, a number of new studies have appeared reporting that hypnotic usage is related to cancer incidence and mortality. Hartz and Ross found a significant association of hypnotic use with melanoma and close-to-significant associations for lung and breast cancers ^72^. Kao *et al.* found a remarkable 6.24 (4.13–9.43, 95% CI) hazard ratio for cancer incidence among those using at least 300 mg of zolpidem per year without other-benzodiazepine consumption (this would correspond to slightly more than one 5 mg dose per week) ^73^. In this Taiwanese national study, smoking and body mass index (BMI) were not controlled, but the overall cancer hazard ratios for zolpidem users were almost identical among men and women, despite an almost 11-fold greater prevalence of smoking among adult men compared to Taiwanese women at the time ^74^. BMI was not controlled, but at that time in Taiwan, although being overweight was more common among women, obesity was more common among men ^75^. However, hepatocellular carcinoma was not associated with zolpidem use in a case-control study ^76^. In a complementary study of benzodiazepines in Taiwan, benzodiazepines were associated with a 1.19 (1.08–1.32 95% CI) cancer incidence hazard ratio, with over twice the benzodiazepine-associated hazard among men as among women ^77^. Similarly, a brief analysis of the national data from Taiwan found a significant cancer adjusted odds ratio for two of three benzodiazepine hypnotics ^78^.

In the Geisinger Health study using electronic medical records, Kripke *et al.* found a hazard ratio for cancer incidence of 1.35 (1.18–1.55 95% CI) associated with use of >132 hypnotic doses per year, with specific hazard ratios of 1.28 (1.03–1.59) for high-dose zolpidem and 1.99 (1.57–2.52) for high-dose temazepam ^26^. There was a significant dose-response. This study was carefully controlled for age, gender, smoking, BMI, and by matching comorbidities among cases and controls. Jiao *et al.* found no excess of colorectal cancer among those reporting sleeping pill usage <3 times per week versus ≥3 times per week in the Women’s Health Initiative data set ^79^, a result consistent with the Hartz and Ross report on the same data set ^72^, but since the contrast of frequencies of usage was weak and the type and quantity of hypnotic consumption were not determined objectively, the negative observation was not very persuasive. We would not expect hypnotics to promote all cancers equally. Indeed, selective specificity among cancer types would be anticipated if the mechanisms are causal. Pottegard *et al.* and Sivertsen *et al.* found small but significant associations of hypnotic usage with cancer, especially lung cancer ^80, 81^, but since they had not controlled for cigarette smoking, both groups thought their result might have arisen from confounding, albeit confounding was not conclusively demonstrated ^82^. That investigators failed to control for important confounders is not proof that confounding explains the significant hazard. Several U.S. and European groups ^80, 81^ and also Kao *et al.* ^73^ found high hazard ratios for lung and esophageal tumors, and the two San Diego studies had carefully controlled for smoking ^26, 71^. We had proposed that effects of hypnotics on weakening the gastro-esophageal sphincter and permitting more gastro-esophageal regurgitation ^83^ might account for the high cancer-specific rates of esophageal and lung tumors ^26^. A new meta-analysis of epidemiological case-control and cohort studies found an overall cancer hazard ratio of 1.29 (1.08-1.53, 95% CI) ^84^. This meta-analysis found that zolpidem had a higher hazard ratio than benzodiazepines and that particularly high hazard ratios were found for esophagus and lung cancers, among other cancer sites with statistical significance. These multiple studies finding hypnotics associated with human lung cancer were consistent with concerns of FDA scientists about lung cancers found in animal studies of zopiclone. The lung cancer specificity supports causality.

There was one pair of studies that was neither clearly confirmatory nor negative. A large-scale survey screening many drugs with a questionable scheme for reusing controls for multiple tests and incorporating a questionable 2-year drug-to-cancer lag remarked no significant association of cancer with temazepam or zolpidem but did find significant associations with oxazepam and perhaps lorazepam, using P<0.01 and relative risk >1.50 as criteria ^85^. In that study, it was not always possible to control for smoking, and control for other confounders was crude and not well-standardized. A similar study added a possible association for phenobarbital ^86^.

In a formal meta-analysis of 22 prospective cohort studies with 2,482,625 participants suffering 312,203 incident cancers, benzodiazepine drug use (including agonists) was found associated with increased cancer risk “(RR:1.25: 95% CI, 1.15-1.36)”, showing dose-response and dose-duration relationships and specificity among cancer types ^87^.

To summarize the cancer epidemiology, the available clastogenicity data, animal data, randomized placebo-controlled clinical trials, and human epidemiology studies rather consistently, if not always conclusively, suggested that hypnotics likely cause human cancers and cancer deaths.

## Hypnotics increase incidence of clinical depression

In combined clinical trials, participants randomized to hypnotics suffered 2.1 times as many incident (new) depressions as those randomized to placebo (P<0.002) ^34^. These were not exacerbations of pre-existing depressions. These were depressions caused by the hypnotics. There are other data demonstrating worsening of depression with a wider variety of popular benzodiazepine and GABA agonists ^88^. Treatment of insomnia by hypnotics causing comorbid depression stands in marked contrast to cognitive-behavioral treatment of insomnia, that has been shown to decrease comorbid depression ^89, 90^ and otherwise improve mental health ^91^.

Some studies have appeared designed to show that a hypnotic reduced depression scores among patients given an antidepressant known to cause insomnia ^92, 93^. In the first of these studies, the benefit of the hypnotic for depression was not significant at week 4 after the investigators removed the rating scale items related to insomnia, whereas the week 8 benefit was nominally significant only at the P=0.04 level not correcting for multiple comparisons. In other words, using rigorous Bonferroni correction for multiple comparisons, the alleged benefit of hypnotic for depression symptoms was not significant. In a second study the authors more readily conceded that the hypnotic had no significant benefit for depression. These studies failed to rebut the evidence that hypnotics cause new depressions.

Hypnotic use is associated with high rates of suicide ^36, 71, 94^. Depression is the major cause of suicide. Panic attacks are another risk factor for suicide ^88^. Short-acting benzodiazepine agonists such as triazolam and zolpidem may cause withdrawal anxiety and even panic attacks during the daytime ^95^. Suicide has been recently described as the 8 ^th^ or 10 ^th^ leading cause of death in the United States ^96, 97^. Indeed, comprehensive toxicological studies have found intoxicating abusable substances (mainly sedative-hypnotics) in a majority of suicides, often combined with alcohol in 30–40% ^88^. Suicides due to overdoses have increased dramatically from 1999 to 2010 in the U.S. ^98^, but there have been an even larger number of deaths of undetermined manner in which suicide through overdose must be suspected ^99^. A very recent report estimated that in 2013 there were 7,000 overdose deaths related to anxiety and sleep medications ^97^, but this did not include all suicides in which the most rigorous toxicology shows a sedative or anxiolytic often mixed with alcohol to be present ^88^. The adjusted odds rate for suicide was 4.2 among hypnotic users as compared to nonusers in one study of elderly people, whereas the odds were not elevated among anti-depressant users (tending to exclude depression and other comorbidities as confounders ^100^.) Prescription sleeping pill use was a stronger predictor of suicide attempts than insomnia symptoms in the National Comorbidity Survey Replication ^101^. In a large study from Taiwan, the adjusted suicide hazard ratio for “needing sleeping pills” was 11.1, whereas the hazard ratio for those reporting sleeping only 0–4 hours adjusted for sleeping pill use was only 3.5, and none of the hazard ratios for insomnia symptoms exceeded 2.0 ^102^. Another national Taiwan study found increased suicides and attempts associated with zolpidem ^94^. The findings indicate that the association of suicides with hypnotic use cannot be entirely attributed to confounders with reverse causality, since the association of hypnotic usage with depression is known to be largely caused by the hypnotics ^34^. Since the genetic influences promoting insomnia and depression appear highly correlated ^103^, the associations both of depression and insomnia with mortality may be mediated through hypnotic drug consumption.

Zolpidem specifically has been implicated as a causal agent in a number of suicides, some of which involved kinds of dissociative behavior often attributed to zolpidem or to combined use of zolpidem with other drugs or alcohol ^104^. Impairments of cognition and judgment that may be caused by sleeping pills ^105^ as well as hallucinations ^106^, irrational behaviors ^98, 107– 109^, and behavioral disinhibition ^88^ may all contribute to suicides, violence, and accidents, even among people who are not severely depressed. However, preliminary results of a recent study listed at https://clinicaltrials.gov/ct2/show/results/NCT01689909 suggested that 8 weeks treatment with zolpidem 10 mg. may have reduced suicidal ideation among patients treated with an SSRI antidepressant.

An authoritative review documented overwhelming evidence of the association of hypnotics with suicide but discerned no evidence of causality ^110^. However, new evidence shows that major components of depression and suicide are linked to infections. Those with inflammation indicated by high C-reactive protein (CRP) had more depression and bipolar disorder and more than twice the suicide rate of those with low CRP ^111– 113^. A Mendelian randomization study proved that CRP has a causal role ^111^, though elevated TNF-alpha, interleukins, and other parts of the immune system may also be factors ^114– 116^. Since it is known that hypnotics cause infections that cause inflammation, a causal pathway from hypnotics to depression and suicide has been demonstrated.

## Automobile crashes, falls, and other accidents are associated with hypnotics

Accidents of all sorts are associated with use of benzodiazepines and benzodiazepine agonists such as zolpidem ^117– 119^. Hypnotic drugs impair next-day alertness, motor skills, reasoning, and overall performance. Most hypnotics impair automobile driving, as indicated by on-the-road controlled performance testing ^120^. This impairment in some instances exceeds the impairment produced by a blood alcohol concentration of 0.05% ^121^. Drivers’ ability to predict their own impairment is poor ^122^. The use of hypnotics and other sedatives is strongly associated with driver hospitalization ^123^ and on-the-road driver-at-fault crashes ^124– 129^. In addition to accidents attributable to impaired coordination, impaired motor skills and loss of alertness, hypnotics may also lead to fatal crashes due to drug induced suicidal thinking, impaired judgment, or recklessness on the part of intoxicated drivers ^96^. Hypnotics are a factor in more than half of intoxication and dangerous driving deaths.

Some crashes result in deaths of passengers and other-vehicle occupants not themselves using hypnotics, but non-driver deaths are not attributed to the hypnotics on death certificates. One study found that use of benzodiazepines and "Z" hypnotics was increased among victims of homicide as well as among the homicide perpetrators ^130^. Thus, both through bad driving and homicides, hypnotics result in deaths that have not been accounted directly as deaths from these hypnotic drugs.

It is well known that falls and accidental injuries are strongly associated with hypnotic usage, in particular hip fractures among aging patients ^131– 141^. Hip fracture is a sometimes-lethal injury. The preponderance of studies indicates a true association of the use of hypnotics and falls, that is thought to be due to the properties of benzodiazepine agonists in inhibiting psychomotor skills and in causing weakness, slowed reflexes, and impaired judgment, especially less than 8 hours after ingestion. After taking a hypnotic at bedtime, older people may get up during the night, e.g., to visit the bathroom, when the pharmacologic impairment from a hypnotic is near-maximum and is combined with impairments from sleepiness and the low point in the biological rhythm of performance. An interesting new systematic review observed that the risk of hip fracture was higher with short-term than long-term use of benzodiazepines and Z-drugs, suggesting that the risk may be greatest before adaptation and tolerance develop ^142^.

A nursing-home study challenged these conclusions, arguing that it was insomnia, not hypnotics, that was associated with falls. This study did not appear to control for confounding sleep apnea, Alzheimer’s disease, or cognitive-behavioral disorders ^143^. It should be conceded that confounders are likely have some influence on risk ratios associating hypnotics with accidental injuries, but the scientific consensus suggests that the association is nevertheless partly causal, based in part on controlled trials showing hypnotic impairments of driving and other forms of psychomotor performance. A causal element is inferred by the majority of authorities.

## Safe doses of hypnotics for target populations are unknown

Animal studies indicate that some individuals in an animal research sample may succumb to a lethal hypnotic-drug effect at doses as low as one-fifth that which is universally lethal ^41^. Variations in susceptibility in a human population varying in age, gender, genetics, and health status is likely to be greater than that in a sample of laboratory animals. The minimum lethal dose of hypnotic drugs in humans is unknown, that is, the dose that might produce fatal respiratory arrest in one person out of 1000 in a representative population or one in 10,000. So many billions of hypnotic doses are prescribed yearly in the U.S. that one death per 10,000 doses would yield over 100,000 deaths per year. Moreover, there are no human dose-response data and very little animal data concerning what doses of hypnotics may be lethal in the presence of opiates, other sedatives, alcohol, aging, obesity, COPD, and other comorbidities. Yet most recognized hypnotic-related deaths are observed in the presence of such additional factors. More study is needed to establish safe doses of hypnotics (if any) when taken with other medications and in the presence of potential comorbidities. As for aging, the consensus of the American Geriatrics Society is that hypnotics are not safe for elderly patients in any dose ^144^.

## Contributory factors combined with hypnotics could cause covert deaths

There is a vast discrepancy between the hundreds of thousands of yearly hypnotic-associated deaths implied by the high epidemiologic hazard ratios and the mere thousands of yearly death certificates in which a hypnotic is listed among the causes of death. Below are presented some of the possible explanations for this discrepancy.

### Obesity and aging exacerbate hypnotic risks

Obesity and aging are perhaps the two most important risk factors for sleep apneas, that is, brief cessations of breathing during sleep ^145^. Sleep apneas occurs at least a few times per hour in the majority of adults over age 40 years and in a great majority of those over age 65 ^145, 146^. If the duration of a sleep apnea before arousal becomes excessively prolonged, e.g., by a hypnotic, death could result. Thus, hypnotic-related hazard ratios are higher among obese patients (see Geisinger Health study supplement Tables 2 and 7 ^26^.) Since there is no evidence that the huge increase in hypnotic hazards among obese patients can be attributed overdoses, it appears that obesity predisposes to covert hypnotic-related deaths, probably by prolonging apneas. It is plausible that among susceptible patients, combinations of aging, obesity, sleep apnea, hypnotics, opiates, other sedatives, and alcohol could produce quiet respiratory cessations followed by cardiac cessation and death even without any ingested doses above common medical practice being taken.

### Prescription and non-prescription opiate use increase hypnotic risks

The use of opiates has become increasingly common in recent years ^147^. Opiates are respiratory suppressants that (like pentobarbital) in overdose can produce respiratory arrest and cardiac arrest. Among patients taking both benzodiazepines and opiates, a remarkable 75% were found to have sleep apnea, and causality was suggested by significant dose-response correlations both for the opiates and for the benzodiazepines ^148^. In some patients, this combination of benzodiazepine and opiate causes hypoxemia (low oxygen) ^149^. Our sleep clinic has recorded polysomnographic data from patients who suffered profound almost continuous apnea with severe hypoxemia due to combinations of hypnotics and opiates. Recall that it is understood on a molecular level how benzodiazepine agonists and opiates combine to suppress firing of respiratory neurons that are necessary to breathe. Patients receiving a combination of benzodiazepines and opiates have increased mortality ^10, 150, 151^. The combination of opiates and benzodiazepines has caused a growing overdose problem in emergency rooms ^147^. Moreover, the most serious overdose problems are seen when opiates and benzodiazepines are combined with alcohol in older patients, reflecting combined effects of opiate, benzodiazepine, alcohol, and aging ^10, 15^.

It may be relevant that close to 70% of hospice patients were taking an opiate and an anxiolytic or hypnotic in the last week of life ^152^. This is not evidence by itself whether this combination influences the survival of hospice patients, nor is the author commenting on the ethics of combining such drugs in a genuine hospice situation. However, most patients given hypnotics and opiates combined have not consented to hospice management.

### Quiet deaths from hypnotics with contributory factors go undetected by medical examiners and unrecorded

In combined-sedative deaths, the individual drug concentrations present in blood may appear within customary therapeutic ranges. Even if a patient is undergoing cardio-respiratory monitoring at the time when respiratory cessation followed by cardiac cessation occurs, there is usually no way of determining whether the fatal respiratory cessation was due to hypnotic drugs in combination with various contributory factors. Especially when death occurs quietly at night (for example, death of an elderly obese patient known to have various comorbidities,) there usually is no autopsy. Physicians signing the death certificates may be tempted to list a cardiac event or a stroke or some long-standing comorbidity as the cause of death without recognizing when hypnotic-induced respiratory suppression was the precipitant.

The press described a highly-distinguished example of how cause-of-death data may be unreliable after U.S. Supreme Court Justice Scalia died unexpectedly at night. According to numerous news reports and sheriff’s documents, Justice Scalia’s appearance was that of a person who had peacefully stopped breathing at night. There was no sign of agitation due to cardiac pain, nor had Justice Scalia complained of cardiac symptoms before going to bed. Justice Scalia might have been taking hypnotics and opiates for the jet lag and pain he was known to be suffering when he arrived at a hunting lodge that routinely gives each guest a free bottle of wine. Without ever viewing the deceased or his bedroom, much less determining what hypnotics, opiates for pain, and alcohol Justice Scalia might have consumed, a local official was guided by Justice Scalia's physician (thousands of miles away) to declare heart attack as the cause of death. Without an autopsy, we will never know if this death was precipitated by hypnotics or opiates and alcohol or if there was a heart attack. Even if a physician suspects that a hypnotic had a role, the physician has little motivation to suggest the hypnotic as a cause of death when it would be hard to prove and may reflect negatively on whatever physician prescribed that hypnotic.

Along the same lines, when hypnotics cause infection, cancer, depression, falls or other accidents, or murder, hypnotics are rarely listed among the causes of death. These patterns along with quiet respiratory deaths may explain why epidemiology shows much higher risks of death associated with hypnotics than the death certificates document. Nevertheless, even the numbers documented in death certificates are too high to be acceptable.

### Commonly-prescribed hypnotics are mainly used inappropriately

Zolpidem, reportedly the most commonly-prescribed hypnotic in the U.S., with an estimated 40 million outpatient prescriptions in 2013 ^153^, ranked first for emergency department visits among psychotropic drugs according to CDC data ^153, 154^. According to the Agency for Healthcare Research and Quality (AHRQ) data, 68% of zolpidem patients were sustained users (three or more prescriptions), and of those 22% were also sustained users of opioids ^153^. Note that recent CDC guidelines recommend against use of benzodiazepine agonists with opiates ^155^. Although the FDA now recommends dosing women with only 5 mg or 6.25 mg of zolpidem, at least to begin with, only 5% of women and 10% of elderly were prescribed these low doses in 2012 ^153^. Apparently, there was little change in dosing for women and elderly after the FDA recommended the low doses in 2013 ^153, 156^. Moreover, 23% of patients with sustained use took another drug targeting the same receptors. A high percentage were depressed, as indicated by 34% of sustained zolpidem users also receiving antidepressants ^153^. Similarly, a 1999–2010 compilation of NHANES data found that 48% of those taking an insomnia medication were ≥60 years of age ^157^. Moreover, over half of those who took a pill for insomnia in the past month were alcohol users (most moderate or heavy users), 56% took other sedatives, and 25% used opioids. In the NHANES data, only a minority of the sedatives taken for sleep were insomnia drugs, but most of the remainder were other benzodiazepines. Recall also that the American Geriatrics Society recommended avoidance of any use of hypnotics or benzodiazepines for elderly patients ^144^, though about half of those receiving hypnotics have been elderly. Analyses of recent U.S. national data indicated that 77.4% of zolpidem prescription recipients had ≥2 safety contraindications ^158^.

There is the further problem of hypnotic misuse in addition to the issues that the great majority of hypnotic prescriptions lack the indication of diagnosed insomnia, or have been prescribed despite contraindications, or have been prescribed in excessive dosages or for excessive durations. According the CBHSQ Report of the U.S. Substance Abuse and Mental Health Services Administration, misuse included taking the drug without a personal prescription, or in greater amounts or longer than instructed, or use in any other way a doctor did not direct ^159^. The National Survey on Drug Use and Health estimated that in 2015, over 1.0 million U.S. adults misused prescription sedatives for sleep, and over 1.2 million misused prescription tranquilizers to help with sleep ^159^. A recent French study found that an indicator of “doctor shopping” to obtain zolpidem exceeded doctor shopping to obtain oxycodone and most other opiates ^160^. Combining the evidence of lack of indication, excessive contraindications, and misuse, it appears that fewer than 10% consuming zolpidem were prescribed the drug in accord with the consensus approved circumstances.

Hypnotics may cause death from overdoses of inappropriate drug-combination prescribing as well as other contributory factors, not only from a lethal dosage of the hypnotic considered by itself.

## Hypnotics cause withdrawal insomnia, anxiety, panic, and epilepsy

It has been well known since they came into use over a century ago that hypnotics and similar sedatives are addicting drugs, frequently eliciting tolerance, physical dependence, and withdrawal reactions ^161^. Most of the benzodiazepine agonist hypnotics and even suvorexant are controlled like addicting drugs by the U.S. Drug Enforcement Agency (DEA). Withdrawal from benzodiazepine agonists can cause insomnia, anxiety, agitation, confusion, and panic and even more severe somatic symptoms such as seizures and death in extreme cases ^35, 95, 155, 162^. In addition, some of the short-acting sedatives such as triazolam and zolpidem may sometimes cause anxiety or agitation during the day following administration before the previous bedtime. Dr. Kripke has treated two patients taking triazolam who developed daytime panic attacks that remitted upon triazolam withdrawal and recurred upon re-challenge. There is also evidence that prolonged use of hypnotics may lead to lasting insomnia, as a consequence causing patients who withdrew from hypnotics to sleep worse than patients who had been randomized in parallel clinical trials to placeboes ^35^. How long this withdrawal insomnia might persist has never been adequately defined.

In another example of sedative withdrawal leading to hyperexcitability, there is a report that benzodiazepine use and withdrawal may result in lasting increased epilepsy ^163^.

## Relationship of hypnotics to insomnia, long sleep, and short sleep

A pioneering large epidemiological study that the American Cancer Society conducted over 50 years ago observed an increased risk of death following hypnotic use. The Cancer Prevention Study I (CPSI) obtained questionnaires in 1958 from over 1,000,000 participants and reliably ascertained their death or survival over 6 years ^164^. The data showed that both long and short sleep predicted elevated mortality (with 7 hours associated with minimal mortality for each age group). This study (often replicated) raised scientific doubt whether there is medical value to increasing reported sleep duration of an adult beyond 7 hours, though it also demonstrated that many adults reporting more than 7 hours of sleep were taking sleeping pills. Sleep durations below the population median are partly attributable to inherited traits, so whether there would be any health benefit in sedating people with short sleep durations to sleep longer remains to be demonstrated. A small objective study of sleep duration recorded by wrist activity suggested increased mortality above 390 minutes of actual sleep (which is greater than the current median sleep of American adults studied with similar technology ^165^.) In the CPSI data, self-reported insomnia had little or no additional mortality effect beyond hours of sleep, although insomnia was moderately associated with short sleep. In contrast, reported sleeping pill use was associated with about 50% increased mortality after controlling for age, gender, reported sleep duration, and reported insomnia ^166^. This was statistically a highly significant result in a million participants, but uncertainty about what participants meant by taking “sleeping pills” “Often” in terms of drug type and frequency demanded more study. The American Cancer Society performed a second Cancer Prevention Study (CPSII) with participants completing over 1.1 million questionnaires in the fall of 1982. CPSII used more explicit questions about sleep duration, insomnia, and “prescription sleeping pills.” After controlling simultaneously for 32 covariates and confounders such as insomnia and sleep duration in Cox Proportional Hazards models, results again showed that use of hypnotics was associated with elevated mortality not attributable to major confounders such as cigarette smoking. Indeed, the mortality risk associated with taking “prescription sleeping pills” was surprisingly comparable to that associated with smoking a pack of cigarettes a day ^71^.

More recent meta-analyses have indicated that the mortality risk associated with short sleep is minimal compared to that associated with long sleep ^167, 168^. Several recent studies have replicated the CPSI and CPSII estimates that insomnia has little or no association with mortality after control for confounders ^169, 170^, but not all studies agree. Although a hypothesis that short sleep causes obesity has received recent popularity, some fostered by investigators affiliated with hypnotics manufacturers, no controlled trials indicating that hypnotics reduce obesity have been located. Epidemiologic data imply that hypnotic usage is more strongly associated with obesity than short sleep itself (see Lawman *et al.*, supplement figure B) ^171^. In summary, there is no scientific rationale that health would be improved by giving hypnotics for short sleep.

## Benefits of hypnotics: minimal

### Popular prescribed hypnotics fail objectively to increase sleep significantly even at high doses: new guidelines discourage hypnotic use

In an authoritative National Institutes of Health (NIH)-sponsored meta-analysis of controlled trials including unpublished trials ^172^, Buscemi and colleagues found that although non-benzodiazepine zolpidem-like drugs [“Z-drugs”] shortened sleep onset latency by an average of 12 minutes (9–17 min, 95% CI), according to objective polysomnograms, these hypnotics increased total nightly sleep time by only 11 minutes (-1 to 23 min, 95% CI, NS). That is, these “Z” drugs produced no substantial statistically-reliable increase in total sleep, even at doses higher than currently recommended. Most of the meta-analyzed studies of zolpidem used doses of 10 mg or more (as high as 30 mg) ^172^, and most of the studies of zopiclone used 7.5 mg doses or more (containing more eszopiclone than any dose approved in the U.S.) The FDA-approved recommended initial zolpidem dosage for most patients is now 5 mg (6.25 mg for the sustained-release form ^173^.) Zolpidem and zolpidem-like drugs constitute the bulk of the current U.S. hypnotics market. Based on all available clinical studies, these lower doses would objectively increase sleep by trivial amounts if at all ^153^. Indeed, the primary zolpidem manufacturer advised the FDA that the 5–6.25 mg dosages were generally ineffective ^44^. The newly-recommended 1-mg dosage of eszopiclone is similarly ineffective ^174, 175^ and even 3 mg. was ineffective in a small study ^176^. Patients typically report more increase in sleep than is measured objectively, but even this self-reported “improvement” at above-recommended doses (which is not supported by objective measurement) is a mere 32 minutes (26–38 minutes, 95% CI) ^172^. The discrepancies between objective and patient-subjective data may be attributable to the amnesic properties of hypnotics, erasing patients’ memories of how much time they are awake in bed. In conclusion, the FDA-recommended doses of the most popular benzodiazepine agonists are virtually ineffective for objectively increasing sleep. Older benzodiazepines are not much more effective.

A new Comparative Effectiveness Review sponsored by the U.S. AHRQ has recently examined the *Management of Insomnia Disorder*, largely referring to chronic insomnia ^177^. As a prepublication Peer Reviewer of this report, I was and still remain very critical of its limitation to mainly-subjective data that are known to give a rosier evaluation of hypnotic effects than objective evaluations, its focus on published reports that are known to be commonly biased towards reporting favorable drug results ^178, 179^, and the AHRQ report's incomplete attention to adverse effects. Nevertheless, it was striking that the AHRQ study found that the strongest evidence for treatment efficacy was with the cognitive-behavioral treatment of insomnia. The evidence for short-term efficacy of zolpidem and eszopiclone in high doses was considered less sufficient, and evidence for efficacy of other hypnotics was judged to be almost entirely insufficient. Moreover, by its clinical trial selection criteria, this Review found essentially no evidence for efficacy of the very low doses of zolpidem and eszopiclone currently recommended by the FDA for most patients, because higher doses appeared unsafe to FDA. In short, the AHRQ study presented no reason why hypnotics are needed, since cognitive-behavioral treatment of insomnia is better. The AHRQ Review found evidence for increased adverse effects with hypnotics compared to placebo, including hypnotic adverse effects of concern (their selection of studies highlighted fractures and dementia) ^180^. This means that patients randomly treated with hypnotics tended to develop more illness and symptoms, quite the opposite of promoting health. The Review found mention of adverse effects virtually absent for the cognitive-behavioral treatment studies ^177^. Although the Comparative Effectiveness Review found insufficient studies to estimate the comparative effectiveness of hypnotics versus cognitive-behavioral treatments, when it reviewed potential harms, there was no contest. Moreover, controlled trials reviewed above prove that hypnotics cause comorbidities such as infection and depression and driving impairments, whereas cognitive-behavioral treatment has been found to decrease medical comorbidities such as depression ^89^.

Whatever weak evidence for benefits of hypnotics there has been came mainly from carefully selected groups of patients with diagnosed insomnia and few if any comorbidities or contraindications, and who generally did not use opiates or other sedatives or excess alcohol ^181^. There are no clinical trials data demonstrating benefit among patients with multiple comorbidities and contraindications while lacking diagnosed insomnia, but such vulnerable patients are the majority of patients receiving hypnotics.

Derived from the AHRQ report, A *Practice Guideline* from the American College of Physicians made a still more reserved interpretation of hypnotics’ benefits and risks ^182^. This report advised that cognitive-behavioral treatments should always be the initial treatment for insomnia disorder, and if this therapy was unsuccessful, then short-term use of hypnotics would be questionable. This *Practice Guideline* found the benefits of even short-term hypnotic treatment to be small or trivial and the evidence persuasive for balancing harms. The *Practice Guideline* did not recommend long-term use of hypnotics at all. Going beyond the AHRQ report, that had not systematically investigated the evidence for severe risks, the *Practice Guideline* listed depression as a definite risk and cancer and excess mortality as possible risks, listing the evidence for these harms in considerable detail in its supplement. One wonders if the *Practice Guideline* would have approved use of the particular hypnotics with the most evidence of risks under any circumstances, were the authors aware of the up-to-date severe risk evidence detailed here. The American Geriatric Society and American College of Physicians guidelines were apparently written by experts without substantial financial conflicts. The European Guideline of the European Sleep Research Society, with few co-authors reporting financial conflicts, stated that they largely agreed with American College of Physicians guidelines, specifically favoring CBT over hypnotic treatments, and disapproving any hypnotic treatment beyond 4 weeks ^161^.

A new randomized trial of CBT for insomnia showed that whereas zolpidem 10 mg. taken during the first few weeks added somewhat to benefits of CBT, those continuing on zolpidem slept worse on follow-ups of 6 months, 1 year, and 2 years ^183^. This is one of the first randomized trials with long-enough follow-up to show that in the long run, a hypnotic made sleep subjectively worse.

In contrast, the first author of the 2017 American Academy of Sleep Medicine (AASM) Guideline for Pharmacologic Treatment of Insomnia ^184^ had previously announced an AASM public awareness of insomnia campaign to “partner with a consortium of industry, from which we anticipate (and have already received) considerable financial support ^185^.” The latest AASM Guideline did not disclose whether the AASM was receiving financial support from the hypnotics industry during its preparation, but certainly some of its authors were. Despite conflicting interests, the AASM acknowledged that the evidence for efficacy of any hypnotic was “WEAK” or worse ^184^. The AASM meta-analysis had demonstrably biased focus on sleep benefits of hypnotics, considering that they did not similarly assess risks. The AASM suggested use of the most popular hypnotics with which this review is concerned, even though conceding that they had insufficiently evaluated the less frequent but more serious harms on which this review has focused.

### Hypnotics fail to improve next-day performance or general health

Based on manufacturers’ advertising, patients expect that a hypnotic will improve their function and performance the following day. The truth is just the opposite. In 1982, two sleep experts received support from a hypnotics’ manufacturer to survey the daytime performance literature about hypnotics and found, “Drug-related improvement in performance was not found, and, in comparing active drug to placebo, it is clear that all hypnotics, at some doses, produce decrements in performance the next day ^105^.” Since 1982, the current author has been looking for objective evidence that hypnotics improve the performance of insomnia patients. Decades later, no evidence that GABA-agonist hypnotics improve objective daytime performance in treating insomnia could be located. When there are proven significant effects, the effects are to make performance worse ^120, 186, 187^. To reiterate, neither the AHRQ Comparative Effectiveness Review nor the AASM documented objective evidence of health or functional benefits from hypnotic drugs ^177^. On average, most hypnotics make patients sleepier the next day, not more fully awake.

After 35 years, the author is still looking for any evidence of objective functional benefits. In a letter to *Sleep Medicine*, readers were asked to inform us if “any U.S.-licensed hypnotic ever objectively improved any aspect of insomnia patients’ daytime function or any aspect of general health ^188^.” So far, nobody has informed me of any such evidence.

### Hypnotic drugs are prescribed to patients without valid clinical indication

According to the U.S. National Ambulatory Medical Care Survey, insomnia is a stated reason for a patient’s visit in less than one quarter of office visits where a hypnotic is prescribed ^4^, but for most hypnotics, insomnia was the only approved indication. Moreover, no diagnosis of any sleep disorder at all is made on 35% of office visits when a hypnotic is prescribed, and of the 65% of such patients who are diagnosed with a sleep disorder (such as hypersomnia and most forms of sleep apnea), often a hypnotic would be contraindicated ^4^. Other studies have likewise found that hypnotics are commonly prescribed for patients who have no diagnosis or complaint of insomnia ^157, 166, 189, 190^. Hypnotics are routinely being prescribed without any apparent valid indication in as much as three quarters of the cases. Similarly, 46% of patients receiving polypharmacy of CNS drugs (such as benzodiazepines and benzodiazepine agonists) had no pain, insomnia, or mental health diagnosis ^191^. From the data reviewed it appears that in most cases, hypnotics are prescribed despite a specific contraindication such as aging. For example, in the 2015 Beers criteria of the American Geriatrics Society, the hypnotics of concern in this presentation are all listed as drugs to avoid ^144^. It would be fanciful thinking to imagine that addicting hypnotics could be generally beneficial as usually prescribed: that is, without evidence of general health benefit, without indication, and despite specific contraindications.

### Manufacturers misrepresent hypnotic benefits in direct-to-consumer advertising

An instructive example is a 2006 advertisement representing that “[eszopiclone] provides a full night of sleep (7 to 8 hours).” An equivalent claim was made in a 2007 eszopiclone-hypnotic print advertisement titled “Sleep the night and seize the day…A better tomorrow begins tonight.” In the scientific study cited by both advertisements as evidence ^192^, the average sleep of patients receiving eszopiclone 2 mg was 382 minutes (6 hours, 22 min) and for 3 mg, it was 412 minutes (6 hours, 52 min). The clinical results cited did not support the manufacturer’s claims to “a full 7 to 8 hours of sleep,” even though the 2 mg and 3 mg doses then studied were greater than the currently-recommended starting doses.

As for the manufacturer’s advertised benefits of “seizing the day,” and a “better tomorrow,” the eszopiclone manufacturer’s study demonstrated no significant objective improvement in measured next-day daytime performance or accomplishment. Specifically, an objective morning performance test did not demonstrate significantly better performance with eszopiclone than with placebo ^192^.

It is not my intention to imply that misrepresentation in consumer advertising has come only from a single manufacturer. There have been many examples with other hypnotics.

## Summary of benefits, risks, and alternatives

The evidence is clear: the most popular hypnotics offer little to no benefit to patients in recommended doses. The most recent American Academy of Sleep Medicine's Clinical Guideline for Management of Chronic Insomnia ^193^ stated that the primary goals of treatment of insomnia should be to increase sleep quantity and to enhance daytime function. To the contrary, popular hypnotics in recommended doses do not increase objective sleep substantially (if at all,) and for many patients, hypnotics cause substantial objective next-day functional impairment. The specified hypnotics have no known objective benefits for any aspect of general health.

Contrasting with the dubious benefits, the popular benzodiazepine agonists in the U.S. are associated with increased mortality hazards, comparable to the hazards of barbiturates. Medical examiner data document that over 10,000 deaths every year are directly caused by and attributed to hypnotic drugs, and there is substantial evidence that hypnotics cause additional covert respiratory depression, suicides, infection, cancer, accidents, and other disorders that lead to a far larger number of deaths as well as to non-fatal morbidities and suffering. The exact number of deaths caused by hypnotics cannot be estimated from medical examiner data alone ^147^, because most of the deaths produced by hypnotics are covert or indirect due to hypnotic-induced or hypnotic-exacerbated morbidities.

The epidemiologic hypnotic mortality risk is almost comparable to that of cigarette smoking and many-fold greater than the risk to Americans of violent death.

- Hypnotic drugs  300,000–500,000 U.S. deaths per year ^26^
- Cigarettes                       560,000 U.S. deaths per year ^194^
- Murders                           14,196 U.S. deaths in 2013

This presentation has focused primarily on zolpidem, temazepam, eszopiclone, zaleplon, triazolam, flurazepam, quazepam, and barbiturates used for sleep (such as pentobarbital, amobarbital, and secobarbital). These drugs were the focus because each had been shown epidemiologically to be associated with high mortality hazards ^26^. This presentation has not focused on other drugs used as hypnotics, either because the epidemiologic and controlled-trials data have not been sufficient to assess their risks as hypnotics or because these drugs are approved and may be effective for indications other than insomnia. Alternative hypnotics approved for treating insomnia in the U.S. include diphenhydramine, ramelteon, doxepin, and suvorexant. Moreover, other drugs commonly available for sleep include trazodone (off label) and melatonin (unregulated). The advantage of alternative drugs is that their risk-benefits ratios are less clearly known to be unfavorable, but the alternative drugs certainly have serious risks ^140^.

Contrasted to hypnotics, the preferred treatment for insomnia is the cognitive-behavioral treatment of insomnia, which appears to be more effective in the long run, better for comorbidities, and safer ^177^. Cognitive-behavioral therapy can be effectively provided through written materials, internet training programs, and brief group therapies. It has been argued that cognitive-behavioral treatment saves money, compared to hypnotics ^195^.

Less known, circadian rhythm timing disorders often cause the biologic propensity for sleep to be either delayed (causing trouble falling asleep and trouble waking in the morning) or too advanced (causing evening sleepiness and early awakening). It is unclear how often the circadian rhythm timing disorders have a more important role in insomnia than the cognitive-behavioral elements, but one estimate suggests that “eveningness” may be associated with trouble falling asleep in as much as one quarter of the adult population ^196^. When circadian timing issues are important, properly timed bright light treatment can be a safe, effective, and inexpensive non-drug treatment that also has benefits for comorbidities such as depression ^197^. However, more clinical trials are needed to better define the applicability of bright light treatment for insomnia.

## Appendix A: Epidemiologic Studies of the Mortality Risks of Hypnotic Drugs

1) Kronholm, E., Jousilahti, P., Laatikainen, T., Lallukka, T., Peltonen, M., Seppanen, J., and Virta, L. Trajectories in hypnotic use and approaching death: a register linked case-control study. *Sleep Med.* 2018; in press.
2) Kabat, G. C., Xue, X., Kamensky, V., Zaslavsky, O., Stone, K. L., Johnson, K. C., Wassertheil-Smoller, S., Shadyab, A. H., Luo, J., Hale, L., Qi, L., Cauley, J. A., Brunner, R. L., Manson, J. E., and Rohan, T. E. The association of sleep duration and quality with all-cause and cause-specific mortality in the Women's Health Initiative. *Sleep Med.* 2018; **50**(10):48-54.
3) Choi, J.-W., Lee, J., Jung, S. J., Shin, A., and Lee, Y. J. Use of sedative-hypnotics and mortality: A population-based retrospective cohort study. *J Clin Sleep Med.* 2018; **14**(10),1669-1677
4) Mesrine, S., Gusto, G., Clavel-Chapelon, F., Boutron-Ruault, M. C., and Fournier, A. Use of benzodiazepines and cardiovascular mortality in a cohort of women aged over 50 years. *Eur J Clin Pharmacol.* 2018; **74**(11):1475-1484.
5) Sun, Y., Lin, C. C., Lu, C. J., Hsu, C. Y., and Kao, C. H. Association Between Zolpidem and Suicide: A Nationwide Population-Based Case-Control Study. *Mayo Clin Proc.* 2016; **91**(3):308-315.
6) Lan, T. Y., Zeng, Y. F., Tang, G. J., Kao, H. C., Chiu, H. J., Lan, T. H., and Ho, H. F. The use of hypnotics and mortality - A population-based retrospective cohort study. *PLoS One.* 2015; **10**(12), e0145271.
7) Palmaro A, Dupouy J, Lapeyre-Mestre M. Benzodiazepines and risk of death: Results from two large cohort studies in France and UK. *Eur Neuropsychopharmacol* 2015; **25**(10), 1566-1577.
8) Chung, W. S., Lai, C. Y., Lin, C. L., and Kao, C. H. Adverse respiratory events associated with hypnotics use in patients of chronic obstructive pulmonary disease: A population-based case-control Study. *Medicine (Baltimore)* **94**(27), e1110. 2015.
9) Kriegbaum, M., Hendriksen, C. Vass, M., Mortensen, E. L., Osler, M. Hypnotics and mortality—partial confounding by disease, substance abuse and socioeconomic factors? *Pharmacoepidemiol Drug Sa*f 2015; **24**(7):779-783.
10) Pinot J, Herr M, Robine JM, Aegerter P, Arvieu JJ, Ankri J. Does the Prescription of Anxiolytic and Hypnotic Drugs Increase Mortality in Older Adults? *J Am Geriatr Soc* 2015; **63**(6):1263-5.
11) Weisberg DF, Gordon KS, Barry DT, Becker WC, Crystal S, Edelman EJ, Gaither J, Gordon AJ, Goulet J, Kerns RD, Moore BA, Tate J, Justice AC, Fiellin DA. Long-term Prescription of Opioids and/or Benzodiazepines and Mortality Among HIV-Infected and Uninfected Patients. *J Acquir Immune Defic Syndr* 2015; **69**(2):223-33.
12) Nakafero G, Sanders RD, Nguyen-Van-Tam JS, Myles PR. Association between benzodiazepine use and exacerbations and mortality in patients with asthma: a matched case-control and survival analysis using the United Kingdom Clinical Practice Research Datalink. *Pharmacoepidemiol Drug Saf* 2015; **24**(8):793-802.
13) Neutel CI, Johansen HL. Association between hypnotics use and increased mortality: causation or confounding? *Eur J Clin Pharmacol* 2015; **71**(5):637-42.
14) Frandsen R, Baandrup L, Kjellberg J, Ibsen R, Jennum P. Increased all-cause mortality with psychotropic medication in Parkinson’s disease and controls: a national register-based study. *Parkinsonism Relat Disord* 2014; **20**(11):1124-8.
15) Weich S, Pearce HL, Croft P, Singh S, Crome I, Bashford J, Frisher M. Effect of anxiolytic and hypnotic drug prescriptions on mortality hazards: retrospective cohort study. *BMJ* 2014; **348**:g1996.
16) Chen H-C, Su T-P, Chou P. A 9-year Follow-up Study of Sleep Patterns and Mortality in Community-Dwelling Older Adults in Taiwan. *Sleep* 2013; **36**(8):1187-98.
17) Gunnell D, Chang SS, Tsai MK, Tsao CK, Wen CP. Sleep and suicide: an analysis of a cohort of 394,000 Taiwanese adults. *Soc Psychiatry Psychiatr Epidemiol.* 2013 Apr 2; **48**:1457-65.
18) Jaussent I, Ancelin ML, Berr C, Peres K, Scali J, Besset A, Ritchie K, Dauvilliers Y. Hypnotics and mortality in an elderly general population: a 12-year prospective study. *BMC Med* 2013; **11**(1):212.
19) Obiora E, Hubbard R, Sanders RD, Myles PR. The impact of benzodiazepines on occurrence of pneumonia and mortality from pneumonia: a nested case-control and survival analysis in a population-based cohort. *Thorax* 2012; **68**(2):163-70.
20) Hartz A, Ross JJ. Cohort study of the association of hypnotic use with mortality in postmenopausal women. *BMJ Open* 2012;2:pii: e001413. doi: 10.1136/bmjopen-2012-001413.
21) Kripke DF, Langer RD, Kline LE. Hypnotics’ association with mortality or cancer: a matched cohort study. *BMJ Open* 2012;2(1):e000850.
22) Gisev N, Hartikainen S, Chen TF, Korhonen M, Bell JS. Mortality associated with benzodiazepines and benzodiazepine-related drugs among community-dwelling older people in Finland: a population-based retrospective cohort study. *Can J Psychiatry* 2011; **56**(6):377-81.
23) Rod NH, Vahtera J, Westerlund H, Kivimaki M, Zins M, Goldberg M, Lange T. Sleep Disturbances and Cause-Specific Mortality: Results From the GAZEL Cohort Study. *Am J Epidemiol* 2010; **173**(3):300-9.
24) Belleville G. Mortality hazard associated with anxiolytic and hypnotic drug use in the national population health survey. *Can J Psychiatry* 2010; **55**(9):558-67.
25) Mallon L, Broman JE, Hetta J. Is usage of hypnotics associated with mortality? *Sleep Med* 2009; **10**(3):279-86.
26) Winkelmayer WC, Mehta J, Wang PS. Benzodiazepine use and mortality of incident dialysis patients in the United States. *Kidney Int* 2007; **72**(11):1388-93.
27) Hublin C, Partinen M, Koskenvuo M, Kaprio J. Sleep and mortality: a population-based 22-year follow-up study. *Sleep* 2007; **30**(10):1245-53.
28) Hoffmann VP, Dossenbach M, West TM, Lowry AJ. Mortality in a cohort of outpatients with schizophrenia: 3-year outcomes from the Intercontinental Outpatient Health Outcomes Study (IC-SOHO). *Biol Psychiatry* **61**(8S):163S-164S. Accessed 2007.
29) Hausken AM, Skurtveit S, Tverdal A. Use of anxiolytic or hypnotic drugs and total mortality in a general middle-aged population. *Pharmacoepidemiol Drug Saf* 2007; **16**(8):913-8.
30) Fukuhara S, Green J, Albert J, Mihara H, Pisoni R, Yamazaki S, Akiba T, Akizawa T, Asano Y, Saito A, Port F, Held P, Kurokawa K. Symptoms of depression, prescription of benzodiazepines, and the risk of death in hemodialysis patients in Japan. *Kidney Int* 2006; **70**(10):1866-72.
31) Lack LC, Prior K, Luszcz M. 708. Does insomnia kill the elderly? *Sleep* **29**[Abstract Supplement], A240. Accessed 2006.
32) Phillips B, Mannino DM. Does insomnia kill? *Sleep* 2005; **28**(8):965-71.
33) Ahmad R, Bath PA. Identification of risk factors for 15-year mortality among community-dwelling older people using Cox regression and a genetic algorithm. *J Gerontol A Biol Sci Med Sci* 2005; **60A**:1052-8.
34) Mallon L, Broman J-E, Hetta J. Sleep complaints predict coronary artery disease mortality in males: a 12-year follow-up study of a middle-aged Swedish population. *J Int Med* 2002; **251**:207-16.
35) Hedner J, Caidahl K, Sjoland H, Karlsson T, Herlitz J. Sleep habits and their association with mortality during 5-year follow-up after coronary artery bypass surgery. *Acta Cardiol* 2002; **57**(5):341-8.
36) Kripke DF, Garfinkel L, Wingard DL, Klauber MR, Marler MR. Mortality associated with sleep duration and insomnia. *Arch Gen Psychiatry* 2002; **59**(2):131-6.
37) Kripke DF, Klauber MR, Wingard DL, Fell RL, Assmus JD, Garfinkel L. Mortality hazard associated with prescription hypnotics. *Biol Psychiatry* 1998; **43**(9):687-93.
38) Merlo J, Ostergren PO, Mansson NO, Hanson BS, Ranstam J, Blennow G, Isacsson SO, Melander A. Mortality in elderly men with low psychosocial coping resources using anxiolytic-hypnotic drugs. *Scand J Public Health* 2000; **28**(4):294-7.
39) Sundquist J, Ekedahl A, Johansson S-E. Sales of tranquillizers, hypnotics/sedatives and antidepressants and their relationship with underprivileged area score and mortality and suicide rates. *Eur J Clin Pharmacol* 1996; **51**:105-9.
40) Hays JC, Blazer DG, Foley DJ. Risk of napping: excessive daytime sleepiness and mortality in an older community population. *J Am Geriatr Soc* 1996; **44**:693-8.
41) Merlo J, Hedblad B, Ogren M, Ranstam J, Ostergren PO, Ekedahl A, Hanson BS, Isacsson SO, Liedholm H, Melander A. Increased risk of ischaemic heart disease mortality in elderly men using anxiolytics-hypnotics and analgesics. *Eur J Clin Pharmacol* 1996; **49**:261-5.
42) Brabbins CJ, Dewey ME, Copeland RM, Davidson IA, McWilliam C, Saunders P, Sharma VK, Sullivan C. Insomnia in the elderly: Prevalence, gender differences and relationships with morbidity and mortality. *Int J Ger Psych* 1993; **8**:473-80.
43) Thorogood M, Cowen P, Mann J, Murphy M, Vessey M. Fatal myocardial infarction and use of psychotropic drugs in young women. *Lancet* 1992; **340**:1067-8.
44) Isacson D, Carsjo K, Bergman U, Blackburn JL. Long-term use of benzodiazepines in a Swedish community: an eight-year follow-up. *J Clin Epidemiol* 1992 Apr; **45**(4):429-36.
45) Rumble R, Morgan K. Hypnotics, sleep, and mortality in elderly people. *J Am Geriatr Soc* 1992; **40**:787-91.
46) Kripke DF, Simons RN, Garfinkel L, Hammond EC. Short and long sleep and sleeping pills: Is increased mortality associated? *Arch Gen Psychiatry* 1979; **36**(1):103-16.
