# Supplementary material for: Hypnotic drug risks of mortality, infection, depression, and cancer: but lack of benefit
Source: F1000Res. 2018 Nov 12;5:918. Originally published 2016 May 19. [Version 3] doi: 10.12688/f1000research.8729.3 (PMC4890308; doi:10.12688/f1000research.8729.3)
Supplement: Peer review history

# Peer review: Referee response for version 3

**DOI:** 10.5256/f1000research.18371.r21086

**Published:** 2018-11-13

**Authors:** Barbara A. Phillips

**Affiliations:** 1 Division of Pulmonary, Critical Care, and Sleep Medicine, Good Samaritan Hospital, University of Kentucky, Lexington, KY, USA

This update is on target, appropriate and approved by me.

I have read this submission. I believe that I have an appropriate level of expertise to confirm that it is of an acceptable scientific standard.

# Peer review: Referee response for version 3

**DOI:** 10.5256/f1000research.18371.r21087

**Published:** 2018-11-13

**Authors:** Jerome Siegel

**Affiliations:** 1 Department of Psychiatry and Biobehavioral Sciences, Brain Research Institute, University of California, Los Angeles, Los Angeles, CA, USA

No further comments.

I have read this submission. I believe that I have an appropriate level of expertise to confirm that it is of an acceptable scientific standard.

# Peer review: Referee response for version 2

**DOI:** 10.5256/f1000research.11965.r21113

**Published:** 2017-03-17

**Authors:** Jerome Siegel

**Affiliations:** 1 Department of Psychiatry and Biobehavioral Sciences, Brain Research Institute, University of California, Los Angeles, Los Angeles, CA, USA

I approve the revised manuscript.

I have read this submission. I believe that I have an appropriate level of expertise to confirm that it is of an acceptable scientific standard.

# Peer review: Referee response for version 1

**DOI:** 10.5256/f1000research.9393.r14033

**Published:** 2016-05-27

**Authors:** Barbara A. Phillips

**Affiliations:** 1 Division of Pulmonary, Critical Care, and Sleep Medicine, Good Samaritan Hospital, University of Kentucky, Lexington, KY, USA

This report is important and over due, and most likely would not be published in a journal that accepts advertising from pharmaceutical companies. The author is careful NOT to confuse association with causation. References are complete and up-to-date.

I have read this submission. I believe that I have an appropriate level of expertise to confirm that it is of an acceptable scientific standard.

# Peer review: Referee response for version 1

**DOI:** 10.5256/f1000research.9393.r13914

**Published:** 2016-05-23

**Authors:** Jerome Siegel

**Affiliations:** 1 Department of Psychiatry and Biobehavioral Sciences, Brain Research Institute, University of California, Los Angeles, Los Angeles, CA, USA

This paper pulls together evidence from Dr. Kripke’s own work and subsequent work, which indicate that the use of benzodiazepines and perhaps other sleeping pills is causing thousands, perhaps hundreds of thousands of deaths annually in the United States. He reviews the complete lack of evidence for any positive health effect of the use of these drugs.  This is especially striking because drug companies sponsor a considerable amount of research on their sleeping pills and would undoubtedly publicize any data indicating positive health or lifespan effects – but there do not appear to be any. Kripke also points out the effectiveness of cognitive behavioral therapy for insomnia. This well studied treatment is less expensive, without any known deleterious effects on lifespan or health and produces a long-lasting reduction in insomnia. The effectiveness of cognitive behavioral therapy in the treatment of insomnia contrasts with the miniscule (0-20 min) increase in sleep time produced by sleeping pills, followed by a considerable withdrawal effect if the patient stops taking the pills.   

 Minor suggestions include the following:

1. I would delete the discussion of Judge Scalia’s death. Although it gets one’s attention, without knowing what Scalia was taking and without any documentation of the cause of death, it does more harm than good to the impact of the paper.
2. On page 10, I would delete the paragraph on prescriptions without valid clinical indication. I guess that in many cases the prescribing physician would just say he forgot to document the need. I do not doubt that Dr. Kripke is bringing attention to an important issue, but it is not persuasively presented, in contrast to the rest of his argument.
3. Small typo under “Obesity and aging exacerbate hypnotic risks:”  “can be attributed overdoses “

I have read this submission. I believe that I have an appropriate level of expertise to confirm that it is of an acceptable scientific standard.

# Response

**Published:** 2016-05-30

**Authors:** Daniel F. Kripke

**Affiliations:** University of California, San Diego, USA

The kind reviews and useful contributions from Dr. Siegel and Dr. Phillips are much appreciated.

 Regarding the paragraph about the death certificate of Justice Scalia, debate about his cause of death and concern about the lack of autopsy received considerable press attention in major media in the United States. The process by which a rural judge decided what cause of death to record on the death certificate was uniquely well documented by the press. This illustrated how a death that could have been due to an overdose might not be explored and the overdose possibility might not be recorded.  Knowing what the patient’s primary doctor had or had not prescribed would not resolve the issue of what drugs were or were not taken. This paragraph was intended to exemplify how we may indeed lack adequate documentation of the real cause of death when the plausible possibility of death caused by a hypnotic is not acknowledged on a death certificate.

 Regarding hypnotic prescriptions without a recorded diagnosis of insomnia, indeed the prescribing physician might just say that forgetting to document the insomnia was an oversight, but it is implausible that oversight is the explanation for such a large percentage of total hypnotic prescriptions. If lack of indication is usually an oversight, where is the proof? When I was a medical student in the 1960’s, I was trained that a hypnotic drug should be part of preprinted routine admission orders, and I have verified that routine admission orders for hypnotics are still preprinted in distinguished academic training hospitals in 2016. If we are training young doctors to prescribe a hypnotic without asking the patient whether that patient is experiencing trouble sleeping and without weighing the benefits and risks for the individual, it is plausible that habit persists in primary care. My impression is that prescribing doctors often do not ascertain that the patient has diagnostic criteria for insomnia, and in many cases, physicians know that the patient has no trouble sleeping. The physician might be trying to treat depression or to supplement opiates, but both uses are contraindicated. The physician might be treating some condition further afield such as hypertension or might be intentionally trying to achieve a placebo effect. There were studies documenting such practices several decades ago (references 126 and 136), but I know of no adequate study of 21 ^st^ century U.S. outpatient hypnotic prescribing intentions.  The manufacturers of both zolpidem and suvorexant have informed the FDA that the currently-FDA-mandated recommended doses are ineffective. It is tempting to infer that the FDA countenances the use of ineffective hypnotic doses as placebo implements of the bedside manner, without evidence that benefits outweigh risks of potentially addicting or lethal placeboes.
